# The complete mental health of Australia’s adolescents and emerging adults: distress and wellbeing across 3 nationally representative community samples

**DOI:** 10.1017/S2045796025000083

**Published:** 2025-03-07

**Authors:** Dimity Crisp, Debra Rickwood, Richard Burns, Emily Bariola

**Affiliations:** 1 Discipline of Psychology, University of Canberra, Canberra, ACT, Australia; 2 National Centre for Epidemiology and Population Health, Australian National University, Canberra, Australia; 3 Kantar Public, Melbourne, VIC, Australia; 4 Orima Research, Collingwood, VIC, Australia

**Keywords:** adolescents, community mental health, mental health, population survey

## Abstract

**Aims:**

The high level of psychological distress in young people is a growing concern. However, there are few national surveys that describe the trajectories of mental health and wellbeing through adolescence into early adulthood. Further, existing research has largely focused exclusively on mental ill-health, with little focus on positive mental health. This study provides the first national profile of the mental health and wellbeing of Australians aged 12–25 years.

**Methods:**

Participants completed the National Youth Mental Health survey in 2018 (n_1_ = 3832), 2020 (n_2_ = 974) or 2022 (n_3_ = 961). We applied Keyes’ Complete Mental Health (CMH) framework to derive categories of mental health and wellbeing, and examine rates of CMH over time, by age and gender.

**Results:**

While approximately half of those surveyed reported flourishing (high wellbeing without mental illness), rates of flourishing declined between 2018 and 2022. Rates of flourishing generally decreased with age, and flourishing was more prevalent amongst males than females.

**Conclusions:**

The findings provide a unique contrast of youth mental health pre-, during and post- the COVID-19 pandemic. While rates of psychological distress are consistently high, the proportion of youth reporting flourishing highlights the need to consider all aspects of psychological functioning to accurately understand and respond to the mental health needs of young people.

There is a considerable literature devoted to exploring the mental health of adolescents and young adults. But the extent to which poor mental health and high levels of non-specific psychological distress are a growing concern for young people in Australia and internationally requires further investigation. Commonly assessed in population-based surveys, non-specific psychological distress refers to a combination of cognitive, emotional and psychophysiological symptoms that appear elevated across a range of mental disorders (Kessler *et al.*, 2002). Over the past 15 years, and accentuated by the COVID-19 pandemic, increases in the reported prevalence of psychological distress and mental ill-health amongst adolescents and young adults have been reported both in Australia and internationally (Botha *et al.*, 2023; Brennan *et al.*, 2021; Burns *et al.*, 2020; Daly, 2022; Dharmayani and Mihrshahi, 2025; Enticott *et al.*, 2022; Halladay *et al.*, 2024; Slade *et al.*, 2024; Twenge *et al.*, 2019). The most recent 2020–2022 Australian National Study of Mental Health and Wellbeing (NSMHW) survey reported 25.7% of people aged 16–24 years had experienced high or very high psychological distress in the 30 days prior to the interview, with this proportion higher among females (34.2%) than males (18%) (Australian Bureau of Statistics, 2023). Results from the 2022 annual Mission Australia Youth Mental Health Survey similarly found the proportion of young people aged 15–19 years responding to their survey who reported psychological distress increased from 18.7% in 2012 to 28.8% in 2022, dropping back to 24.9% in 2023, with females more likely to report psychological distress than males (Brennan *et al.*, 2021; Leung *et al.*, 2022; McHale *et al.*, 2023). While international data has also reported increases in distress for younger adolescents (aged 12–17 years) (Daly, 2022; Halladay *et al.*, 2024; Twenge *et al.*, 2019) limited comparable data has been published on younger Australians aged 12–15 year. The 2013–2014 Australian Child and Adolescent Survey of Mental Health and Wellbeing estimated that approximately 20% of adolescents aged 11–17 years had experienced high to very high psychological distress in the past year; with the proportion of females reporting very high psychological distress substantially higher than that of males (Lawrence *et al.*, 2015). However, the extent to which this may have increased is unknown.

Comparing the prevalence rates for Australia, it appears that reported psychological distress may be similar for adolescents and young adults; however, due to the differences in the methodologies applied in each survey, it is not possible to draw clear conclusions about age differences and cohort effects. Notably, the Mission Australia’s Youth Mental Health Survey is distributed via schools, community and youth service providers and organisations, and is not designed to be representative (Mission Australia & Black Dog Institute, 2017). Further, the NSMHW employs face-to-face interviews for data collection, where the Child and Adolescent Survey and Mission Australia Youth Mental Health Survey involved self-report to an online survey on a private tablet device. Research shows that young people may be more likely to disclose sensitive information via technology rather than face-to-face (Bradford and Rickwood, 2015). Finally, while those studies that utilise longitudinal data (e.g. Botha *et al.*, 2023; Burns *et al.*, 2020) are able to point to cohort effects and developmental trends, they do not capture the entire period of adolescent development. Consequently, a nationally representative survey with consistent methodology across the entire 12–25-year age range, conducted at different time periods, is needed to understand age-related trends and provide a complete profile of the mental health of young Australians.

A major limitation to our understanding of the mental health of young Australians has also been the exclusive focus on mental ill-health in Australia’s national surveys, with little or no attention to positive mental health and wellbeing. Acknowledging that vast conceptualisations of wellbeing exist (Marsh *et al.*, 2020; Martela and Sheldon, 2019), for the purpose of the current paper, wellbeing has been conceptualised in line with the Complete Mental Health (CMH) model (Keyes, 2002; Keyes and Lopez, 2002). Developed from the Mental Health Continuum or Dual Continua model (Keyes, 2002, 2005) that recognises psychopathology and wellbeing as distinct dimensions of mental health, the CMH model conceptualises positive mental health and wellbeing as reflective of subjective, psychological and social wellbeing. Positive wellbeing reflects dimensions of subjective, psychological and social wellbeing (Keyes and Lopez, 2002). Subjective wellbeing reflects the presence and absence of positive and negative emotional states (hedonic wellbeing) and life satisfaction (Diener, 1984; Keyes *et al.*, 2002; Ryff *et al.*, 2021). Psychological (eudaimonic) wellbeing encompasses dimensions of positive relations with others, self-acceptance, personal growth, environmental mastery, autonomy, and purpose in life (Ryff *et al.*, 2021). Social wellbeing specifically recognises the importance of social factors such as social integration and connection to the community; social acceptance and perceived trust of others; social contribution (the extent to which individuals believe they are valued members of society); and social actualisation (the belief in society improvement) (Keyes, 1998). Examining positive mental health alongside mental ill-health may help to provide a more complete picture of the mental health status of young people. Studies that have examined the wellbeing of young people are often derived from very small samples or are otherwise limited in national representativeness due to recruitment methodology (Bourke and Geldens, 2007; Hunter *et al.*, 2015). Further research is needed to examine the interplay of both mental wellbeing and mental ill-health components and provide a more comprehensive understanding of people’s mental health status.

Categorisation within the CMH model enables classification of individuals as flourishing, languishing, or as having moderate mental health (Keyes, 2002; Keyes and Lopez, 2002). Each of these categories may then be accompanied by mental illness (Keyes, 2002; Keyes and Lopez, 2002; Venning *et al.*, 2013). Specifically, *Flourishing* reflects the absence of mental illness, with high levels of positive wellbeing. *Languishing* reflects the absence of mental illness, but with low levels of positive wellbeing. In contrast, individuals reporting risk of mental illness with high levels of positive wellbeing are *Struggling*; whereas individuals reporting risk of mental illness with low levels of positive wellbeing are *Floundering.* While categorisation as having moderate mental health is acknowledged in the CMH model, specific terminology for those with moderate levels of positive wellbeing alongside indicators of mental illness has not been reported. As such we refer to the absence of mental illness with moderate levels of positive wellbeing as *Middling*; and individuals reporting risk of mental illness with moderate levels of positive wellbeing as *Stumbling.*

Although a growing interest is emerging in applying the CMH model to adolescents and young adults, to date, research in this area is limited (see Waigel and Lemos, 2023 for review). Considering poor functioning in the absence of identified ill-health may be important for understanding the challenges some young people face during their formative years. Similarly, reported high distress with good functioning may provide insight into the state of today’s youth, particularly in developed countries where protective factors are generally high. Research has shown that flourishing (high wellbeing) can moderate associations between drug use and common mental health conditions such as depression and anxiety (Butler *et al.*, 2019), and is associated with better psychosocial and physical functioning (Moore *et al.*, 2019; Singh *et al.*, 2015; Suldo and Shaffer, 2008). Flourishing has also been associated with higher academic achievement in university, more successful academic self-regulation and better academic engagement (Datu, 2018; Howell, 2009; Moore *et al.*, 2019; Suldo and Shaffer, 2008; Suldo *et al.*, 2011), as well as longer term positive impacts on educational attainment, participation in volunteer activities and civic engagement (O’Connor *et al.*, 2017). In contrast, Languishing has been associated with increased engagement in health-risk behaviours (Venning *et al.*, 2013). Recent research has also suggested that high wellbeing may buffer risk of suicide (Canter *et al.*, 2024; Oh *et al.*, 2024). Specifically, Oh *et al.* (2024) reported that young adults identified as Flourishing were less likely to report suicidal thoughts and behaviours compared to those identified as Languishing or those reporting high wellbeing with depression (identified as Struggling above). Those reporting both depression and low wellbeing (identified as Floundering above) had greatest odds of suicidal thoughts and behaviours. Interestingly, Venning *et al.*’s (2013) investigation of the CMH status of Australian adolescents found the prevalence of Flourishing was lowest amongst older adolescents. Similar age differences have also been indicated in measures of wellbeing (Burke and Minton, 2019). However, further investigation is needed that encompases the adolescent and young adult period of development. Examination of the concept of Flourishing in line with the CMH model, as a combination of both high wellbeing and the absence of mental illness, may offer important insights for targeted interventions for mental health and wellbeing that may need to be age and/or gender specific.

## The current study

The current study provides the first national profile of the CMH of young Australians from 2018 to 2022. The main objective was to determine the proportion of young people aged 12–25 who are Flourishing in comparison to other CMH states (i.e. Middling, Languishing, Struggling, Stumbling or Floundering), and to examine whether these proportions are consistent across age and gender. The study utilises data from three national community surveys of youth mental health and wellbeing conducted in 2018, 2020 and 2022 to examine the experience of young Australians across the entire critical period of adolescence and early adulthood.

## Method

### Participants

Survey 1 comprised 3721, Survey 2 comprised 974, and Survey 3 comprised 961 Australian young people aged 12–25 years from all states and territories in Australia. Table 1 presents the demographic characteristics of each survey. A quota sampling procedure was used, with quotas set for age group, gender, and state/territory to ensure representation as per the demography of the Australian population, and to provide equal representation by age and gender (male/female). While the interview protocol does provide option for participants to indicate their gender as non-binary or gender diverse the representation of this gender category was too small (<2%) to include in the analyses for the present paper. As such only participants identifying as male, or female are reported. Rates of high psychological distress were reported by around 30–40% of the sample; high wellbeing was reported by approximately 60% of the sample.

**Table 1.** Demographic characteristics and mental health state by survey

Demographic	Survey 1	Survey 2	Survey 3
	2018	2020	2022
N	3721	974	961
Age	M = 18.20, SD = 3.99	M = 18.03, SD = 4.03	M = 18.06, SD = 4.05
Gender: female	50.9%	49.7%	49.0%
Aboriginal and/or Torres Strait Islander	2.9%	6.3%	4.2%
Born overseas	10.1%	8.2%	8.6%
Work:			
Employed	53.7%	48.2%	60.4%
Looking for work	16.5%	21.6%	9.3%
NILF and not looking for work	29.8%	30.3%	30.4%
Study:			
Not studying or training	23.6%	24.7%	24.3%
Primary school	1.9%	0.6%	4.0%
Secondary school	46.8%	47.6%	43.8%
University	20.8%	19.6%	19.8%
Vocational education	7.0%	7.4%	8.2%
Sexuality: heterosexual	88.4%	85.1%	82.6%
K10			
Low (10−15)	35.1%	29.5%	20.1%
Moderate (16−21)	34.2%	37.8%	39.2%
High (22−29)	20.7%	24.0%	27.2%
Very high (30−50)	10.0%	8.7%	13.5%
MHC-SF			
Low wellbeing	2.6%	2.9%	3.5%
Moderate wellbeing	35.3%	33.7%	41.8%
High wellbeing	62.1%	63.4%	54.6%
CMH status			
Flourishing^a^	53.0%	52.2%	44.4%
Middling^b^	16.0%	14.7%	14.6%
Languishing^c^	0.3%	0.4%	0.3%
Struggling^d^	9.1%	11.3%	10.2%
Stumbling^e^	19.3%	19.0%	27.3%
Floundering^f^	2.4%	2.5%	3.2%

*Note.* NILF = Not In Labour Force; CMH = Complete Mental Health

a Flourishing = high wellbeing, low risk of mental illness (K10 score < 21).

b Middling = moderate wellbeing, no mental illness (K10 score < 21).

c Languishing = low wellbeing, no mental illness (K10 score < 21).

d Struggling = high wellbeing, at risk of mental illness (K10 score 22+).

e Stumbling = moderate wellbeing, at risk of mental illness (K10 score 22+).

f Floundering = low wellbeing, at risk of mental illness (K10 score 22+).

### Procedure

The study reflects a repeated cross-sectional design. The National Youth Mental Health surveys were conducted in 2018, 2020 and 2022. Each survey recruited an independent sample of young people. Two survey modalities have been used in the conduct of the surveys: a computer assisted telephone interview (CATI) and online survey. In 2018 the predominant mode of data collection was CATI with transition to a combination of CATI and online surveys occurring across 2020 and 2022. Due to potential impact associated with mode of delivery on participant response the data used in the present study reflected only data obtained through CATI, hence differing sample sizes. This removed potential for the differences in mode delivery as an influence on reported distress and wellbeing when comparing across years.

The CATI was administered via social research firm, Colmar Brunton (now Kantar Public), using trained survey interviewers. The sample was drawn via random digit dialing sampling (randomly generating Australian mobile phone numbers and landline numbers). Participants were screened for eligibility (age, gender and location) and provided verbal consent before participating. Consent was also obtained from parents for participants aged 12–17 years. On average, the survey took 30 minutes to complete. Participants received $20 as compensation for participation. Survey 1 was conducted between July and September 2018, Survey 2 between May and June 2020, and Survey 3 between August and September 2022. Ethics approval was obtained from Bellberry Limited Human Research Ethics Committee (2018-05-383, 2020-04-395, 2022-05-526).

### Measures

The measures reported here formed part of a larger questionnaire examining young people’s attitudes and behaviours related to their mental health and wellbeing. *Psychological distress* over the past 4 weeks was measured with the Kessler 10 Psychological Distress Scale (K10) (Kessler *et al.*, 2002). Respondents rated their experience of anxiety and depressive symptoms on a 5-point scale from 1 (*not at all*) to 5 (*all of the time*), total scores ranged from 10 to 50. The scale exhibited high reliability in the current study (2018 α = .89; 2020 α = .88; 2022 α = .88). Based on the scoring protocol used by the Australian Bureau of Statistics (Australian Bureau of Statistics, 2019), scores were categorised as 10–21 low to moderate distress (no mental illness) and 22–50 as high to very high distress (at risk of mental illness). The category of high to very high distress was used as the indicator of risk of mental illness to be consistent with other recent reports of psychological distress in Australian youth (Australian Bureau of Statistics, 2023; Brennan *et al.*, 2021; Leung *et al.*, 2022; McHale *et al.*, 2023).

*Wellbeing* was measured using the Mental Health Continuum – Short form (MHC-SF) (Keyes, 2002). The 14-item scale reflects emotional, psychological and social wellbeing over the past month, with items responded to on 6-point scale from 0 (*never*) to 5 (*every day*). Using the scoring protocol developed by Keyes (2006) participants were categorised as having: high wellbeing if they reported experiencing one or more indicators of subjective wellbeing, and six or more of the indicators of psychological and social wellbeing, every day or almost every day; and low wellbeing if they reported experiencing one or more indicators of subjective wellbeing, and six or more of the indicators of psychological and social wellbeing, either never or only once or twice in the past month. Individuals who did not fall into either of these categories were categorised as having moderate wellbeing. The MHC-SF a very high level of internal consistency in the current surveys (2018 α = .92; 2020 α = .92; 2022 α = .91).

Individuals were categorised into CMH states consistent with approaches used in previous papers (Keyes and Lopez, 2002; Venning *et al.*, 2013; see Table 1). With three levels of mental wellbeing from the MHC-SF and two levels of risk of mental illness established utilising to the K10, six distinct categories of CMH were derived. These are: *Flourishing* (high wellbeing with no mental illness), *Middling* (moderate wellbeing with no mental illness), *Languishing* (low wellbeing with no mental illness), *Struggling* (high wellbeing with risk of mental illness), *Stumbling* (moderate wellbeing and risk of mental illness), *Floundering* (low wellbeing and risk of mental illness).

### Analysis

Data were analysed using STATA 15.1. Listwise deletion was used to retain only participants who were not missing data on the study variables of interest. Owing to small cell sizes in a multi-variable multinomial regression model regressing CMH state on gender, survey and age group factors, models were estimated separately and stratified by survey year. Multinomial regression analyses were used to estimate the differences between gender, age group and survey year on CMH state. Contrasts then examined differences in the probabilities of CMH states between each level of the IVs: gender (male, female), age group (12–14, 15–17, 18–21, 22–25) and survey (2018, 2020, 2022) were conducted using pairwise comparisons of the marginal means. We consider differences reported in the context of the effect sizes and very high probability values (e.g. p < .001; Wasserstein *et al.*, 2019). For the analysis of gender and age groups, analyses were stratified by survey.

## Results

### Survey differences

Table 1 presents the proportion of young people identified within each CMH state across the surveys. Results from a multinomial regression revealed significant differences between surveys in the proportions across the CMH states (χ^2^ (10) = 44.59; p < .001). The 2022 survey indicated a smaller proportion of young people as Flourishing compared to both 2018 (Unadj z = 4.76, p < .001) and 2020 surveys (Unadj z = 3.41, p = .001). In contrast, the 2022 survey reported a higher proportion of young people as Stumbling compared to 2018 (Unadj z = 5.04, p < .001) and 2020 (Unadj z = 4.33, p < .001). A small difference was noted in the proportion of young people Struggling between 2018 and 2020 (Unadj z = 2.00, p = .045).

### Gender differences

Table 2 presents the proportion of males and females for each of the CMH states by survey, with pairwise comparisons of the estimated marginal means. Significant, gender differences in the proportion of young people classified within each of the CMH states were reported across surveys (2018: (χ^2^ (5) = 79.59; p < .001); 2020: (χ^2^ (5) = 21.67; p < .001); 2022: (χ^2^ (5) = 42.32; p < .001)). Pairwise comparisons indicated a significantly higher proportion of males were reported as Flourishing compared to females and a higher proportion of females classified as Stumbling across all surveys. A slightly higher proportion of females were classified as Floundering in 2018, and a slightly higher proportion of females were classified as Struggling in 2020. However, the low prevalence of individuals Floundering makes comparison of differences difficult to quantify. There were no significant differences found in the proportion of males and females classified as Languishing.

**Table 2.** Complete mental health states by year, and gender

CMH state	Males n (%)	Females n (%)	Contrast^b^	SE	p
2018 N	1826	1895			
Flourishing	1065 (58.3)	907 (47.9)	.10	.02	<.001
Middling	315 (17.3)	280 (14.8)	.02	.01	.040
Languishing	<10^a^ (<1.0)	<10^a^ (<1.0)	.00	.00	.953
Struggling	151 (8.3)	186 (9.8)	−.02	.01	.100
Stumbling	257 (14.1)	462 (24.4)	−.10	.01	<.001
Floundering	33 (1.8)	55 (2.9)	−.01	.01	.027
2020 N	490	484			
Flourishing	282 (57.6)	226 (46.7)	.11	.03	.001
Middling	78 (15.9)	65 (13.4)	.02	.02	.272
Languishing	<10^a^ (<1.0)	<10^a^ (<1.0)	.00	.00	.321
Struggling	42 (8.6)	68 (14.1)	−.05	.02	.007
Stumbling	74 (15.1)	111 (22.9)	−.08	.03	.002
Floundering	11 (2.2)	13 (2.7)	−.00	.01	.657
2022 N	490	471			
Flourishing	256 (52.2)	171 (36.3)	.16	.03	.001
Middling	76 (15.5)	64 (13.6)	.02	.02	.398
Languishing	<10^a^ (<1.0)	<10^a^ (<1.0)	.00	.00	.584
Struggling	52 (10.6)	46 (9.8)	.01	.02	.665
Stumbling	91 (18.6)	171 (36.3)	−.18	.03	<.001
Floundering	13 (2.7)	18 (3.8)	−.01	.01	.307

*Note.*

a Cell sizes < 10 are not reported as discrete value.

b Model effect of pairwise comparison of estimated marginal means following multinomial regression analyses.

### Age differences

**Figure 1.**
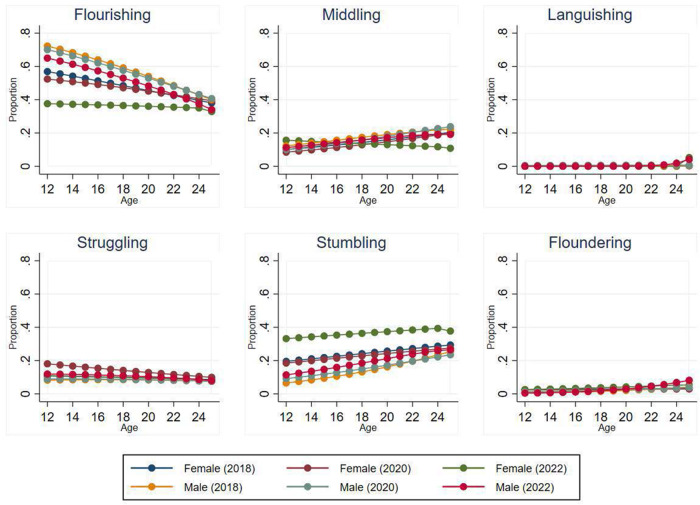
Proportion of young people classified within each complete mental health state across age, by gender and survey.

Age differences in the proportion of individuals classified within each of the CMH states for each survey are presented in Tables 3, 4 and 5, with pairwise comparison of the estimated marginal means. Overall, there were significant differences between age groups in the 2018 (χ^2^ (15) = 176.09.37; p < .001), 2020 (χ^2^ (15) = 39.66; p < .001), and 2022 (χ^2^ (15) = 26.96; p = .029). Pairwise comparisons of the marginal means indicated, a higher proportion of younger adolescents (12-14 years) were classified as Flourishing, with the proportion decreasing with age. In contrast, trends reflect a higher proportion of Stumbling with increasing age, across the surveys. The youngest cohort (12–14 years) were least likely to be classified as Middling in 2018 and 2020. The low prevalence of individuals classified as Languishing and Floundering limits comparison of differences across age for these states. The probabilities of young people classified within each CMH state were plotted (Figure 1) over age by gender and survey. These reveal no overall substantive differences between surveys with the exception of the higher proportion of females indicated as Stumbling in 2022.

**Table 3.** Complete mental health states by age group (survey 1 – 2018)

CMH state	12−14			15−17			18−21			22−25	
	(N = 859)			(N = 948)			(N = 945)			(N = 969)	
	n	%		n	%		n	%		n	%
Flourishing	607	70.7		492	51.9		409	43.3		464	47.9
Middling	86	10.0		141	14.9		188	19.9		180	18.6
Languishing	<10^a^	<1.0		<10^a^	<1.0		<10^a^	<1.0		<10^a^	<1.0
Struggling	80	9.3		93	9.8		80	8.5		84	8.7
Stumbling	81	9.4		192	20.3		231	24.4		215	22.2
Floundering	<10^a^	<1.0		25	2.6		36	3.8		22	2.3
Comparisons			Contrast^b^	SE	p	Contrast^b^	SE	p	Contrast^b^	SE	p
Flourishing		Ref	−.19	.02	<.001	−.27	.02	<.001	−.23	.02	<.001
Middling		Ref	.05	.02	.002	.10	.02	<.001	.09	.02	<.001
Languishing		Ref	.01	.00	.025	.00	.00	.317	.00	.00	.045
Struggling		Ref	.01	.01	.719	−.01	.01	.528	−.01	.01	.631
Stumbling		Ref	.11	.02	<.001	.15	.02	<.001	.13	.02	<.001
Floundering		Ref	.02	.01	<.001	.03	.01	<.001	.02	.01	.002
Flourishing					Ref	−.09	.02	<.001	−.04	.02	.079
Middling					Ref	.05	.02	.004	.04	.02	.030
Languishing					Ref	−.00	.00	.102	−.00	.00	.714
Struggling					Ref	−.01	.01	.310	−.01	.01	.388
Stumbling					Ref	.04	.02	.028	.02	.02	.300
Floundering					Ref	.01	.01	.149	−.00	.01	.604
Flourishing								Ref	.05	.02	.043
Middling								Ref	−.01	.02	.464
Languishing								Ref	.00	.00	.185
Struggling								Ref	.00	.01	.874
Stumbling								Ref	−.02	.02	.243
Floundering								Ref	−.02	.01	.050

*Note.*

a Cell sizes < 10 are not reported as discrete value.

b Model effect of pairwise comparison of estimated marginal means following multinomial regression analyses.

**Table 4.** Complete mental health states by age group (survey 2 – 2020)

CMH state	12−14			15−17			18−21			22−25	
	(N = 238)			(N = 245)			(N = 245)			(N = 246)	
	n	%		N	%		n	%		n	%
Flourishing	156	65.5		121	49.4		122	49.8		109	44.3
Middling	21	8.8		36	14.7		38	15.5		48	19.5
Languishing	<10^a^	<3.0		0	0.0		<10^a^	<3.0		<10^a^	<3.0
Struggling	28	11.8		37	15.1		21	8.6		24	9.8
Stumbling	29	12.2		45	18.4		52	21.2		59	24.0
Floundering	<10^a^	<3.0		<10^a^	<3.0		10	4.1		<10^a^	<3.0
Comparisons			Contrast^b^	SE	p	Contrast^b^	SE	p	Contrast^b^	SE	p
Flourishing		Ref	−.16	.04	<.001	−.16	.04	<.001	−.21	.04	<.001
Middling		Ref	.06	.03	.044	.07	.03	.024	.11	.03	.001
Languishing		Ref	−.00	.00	.316	.00	.01	.578	−.00	.01	.981
Struggling		Ref	.03	.03	.282	−.03	.03	.245	−.02	.03	.476
Stumbling		Ref	.06	.03	.058	.09	.03	.007	.12	.03	.001
Floundering		Ref	.01	.01	.331	.03	.01	.053	.01	.01	.503
Flourishing					Ref	.00	.05	.928	−.05	.04	.259
Middling					Ref	.01	.03	.801	.05	.03	.155
Languishing					Ref	.01	.01	.156	.00	.00	.316
Struggling					Ref	−.07	.03	.025	−.05	.03	.072
Stumbling					Ref	.03	.04	.427	.06	.04	.127
Floundering					Ref	.02	.02	.309	−.00	.01	.755
Flourishing								Ref	−.05	.04	.223
Middling								Ref	.04	.03	.243
Languishing								Ref	−.00	.01	.560
Struggling								Ref	.01	.03	.649
Stumbling								Ref	.03	.04	.465
Floundering								Ref	−.02	.02	.187

*Note.*

a Cell sizes < 10 are not reported as discrete value.

b Model effect of pairwise comparison of estimated marginal means following multinomial regression analyses.

**Table 5.** Complete mental health states by age group (survey 3 – 2022)

CMH state	12−14			15−17			18−21			22−25	
	(N = 238)			(N = 223)			(N = 250)			(N = 250)	
	n	%		N	%		n	%		n	%
Flourishing	130	54.6		100	44.8		96	38.4		101	40.4
Middling	28	11.8		39	17.5		38	15.2		35	14.0
Languishing	0	0.0		0	0.0		0	0.0		<10^a^	<3.0
Struggling	29	12.2		19	8.5		28	11.2		22	8.8
Stumbling	48	20.2		61	27.4		77	30.8		76	30.4
Floundering	<10^a^	<3.0		<10^a^	<3.0		11	4.4		13	5.2
Comparisons			Contrast^b^	SE	p	Contrast^b^	SE	p	Contrast^b^	SE	p
Flourishing		Ref	−.10	.05	.035	−.16	.04	<.001	−.14	.04	.001
Middling		Ref	.05	.03	.082	.03	.03	.265	.02	.03	.461
Languishing		Ref	.00	.00	1.000	.00	.00	1.000	.01	.01	.081
Struggling		Ref	−.04	.03	.195	−.01	.03	.735	−.03	.03	.223
Stumbling		Ref	.07	.04	.070	.11	.04	.007	.10	.04	.009
Floundering		Ref	.01	.01	.641	.03	.01	.034	.04	.02	.013
Flourishing					Ref	−.06	.05	.155	−.04	.05	.329
Middling					Ref	−.02	.03	.502	−.03	.03	.299
Languishing					Ref	.00	.00	1.000	.01	.01	.081
Struggling					Ref	.03	.03	.327	.00	.03	.914
Stumbling					Ref	.03	.04	.409	.03	.04	.465
Floundering					Ref	.03	.02	.097	.03	.02	.040
Flourishing								Ref	.02	.04	.647
Middling								Ref	−.01	.03	.704
Languishing								Ref	.01	.01	.081
Struggling								Ref	−.02	.03	.371
Stumbling								Ref	−.00	.04	.923
Floundering								Ref	.01	.02	.676

*Note.*

a Cell sizes < 10 are not reported as discrete value.

b Model effect of pairwise comparison of estimated marginal means following multinomial regression analyses.

## Discussion

This study aimed to describe the CMH of young Australians by reporting the proportions who were Flourishing, Middling, Languishing, Struggling, Stumbling and Floundering in 2018, 2020 and 2022, and examine any associations between age and gender. Positively, most young people surveyed indicated good mental health status with an absence of likely mental illness and moderate to strong wellbeing protective factors. Around half (44%–53%) were Flourishing, the ideal mental health state, with another 14.6%–16.0% free of distress and with moderate wellbeing (Middling). It is noted that across our samples high rates of psychological distress (approximately 30–40% reported high to very high distress) were reported that exceed that reported by the NSMHW (Australian Bureau of Statistics, 2023) and Mission Australia Youth Mental Health Survey (McHale *et al.*, 2023). However, despite this, only a small proportion of people were found to be Floundering (low wellbeing and risk of mental illness; 2018: 2.5%; 2020: 2.6%; 2022: 3.2%). These results are consistent with other investigations of the CMH classifications amongst adolescent populations (Moore *et al.*, 2019) and highlight that although high distress is reported, many young Australians still report moderate to high wellbeing; emphasising the need to consider all aspects of psychological functioning in order to accurately understand and respond to mental health needs of young people.

Proportions of individuals classified within each of the CMH states were consistent across 2018 and 2020. This result is interesting, particularly in light of the COVID-19 pandemic. While these results indicate little or no immediate impact of the pandemic on the mental health and wellbeing of young Australians, data obtained for the 2020 survey were collected during the initial months of the pandemic in Australia and, while initial public health restrictions were implemented nationally in March 2020, these restrictions were of limited duration and the impacts on mental health and wellbeing may not yet have emerged. While overall increases in mental health problems during the pandemic have been documented globally (see Bower *et al.*, 2023; Wolf and Schmitz, 2024; for reviews), our findings are consistent with other Australian research showing minimal associations between the COVID-19 pandemic and mental health outcomes during the first months of the pandemic (e.g., Batterham *et al.*, 2021; Dawel *et al.*, 2020). The finding that young people in 2022 were less likely to report Flourishing, and more likely to report Stumbling, compared to 2018, and to a lesser extent 2020, may reflect evidence of longer-term impacts of the COVID-19 pandemic on mental health and wellbeing that have seen across other repeated cross-sectional and longitudinal studies of children and adolescents globally (Wolf and Schmitz, 2024).

We found a significantly higher proportion of males classified as Flourishing in all three surveys. These gender differences should not be surprising since females report higher levels of psychological distress (Australian Bureau of Statistics, 2023; Brennan *et al.*, 2021; Leung *et al.*, 2022; McHale *et al.*, 2023), and wellbeing research has found adolescent males to report higher levels of psychological, social and emotional wellbeing than their female peers (Andreou *et al.*, 2020; Burke and Minton, 2019). While it has been proposed that these differences may be associated with the impact of different types of stressors during adolescence and emerging adulthood (e.g., study stress, peer, romantic and family stress, etc.; Andreou *et al.*, 2020), or cultural expectations associated with gender roles (Burke and Minton, 2019), further investigation of the drivers of these differences is needed. However, that no substantive significant gender differences were found in the proportion of young Australians classified as Languishing or as having moderate mental health is notable. It may be that gender differences are only seen at the more extreme categories of the mental health continuum; however, further research in this area is needed.

Consistent with Venning *et al.*’s (2013) investigation of the CMH states within adolescents, and research indicating decreases in levels of wellbeing with increasing age during adolescence (Andreou *et al.*, 2020; Burke and Minton, 2019), we found the proportion of young people classified as Flourishing generally decreased with increasing age into young adulthood. Comparable increases were observed in the proportion of individuals identified as Middling and Stumbling. Together with the relative stability of the other categories this highlights that while there may be small increases in mental ill-health with age, positive wellbeing plays an important role in the functioning of young people, and we need to consider all aspects of psychological functioning to more accurately understand mental health trends.

Developmental changes and the increasing stressors that young people experience as they move from childhood into adolescence and early adulthood are likely primary contributors to decreased Flourishing with age. Research examining emerging adults (aged 18–25 years) has highlighted those changes occurring in areas such as identity, emotion regulation, independence and relationships (Schulenberg *et al.*, 2004) and resultant stressors associated with study, employment, financial pressure, relationships and future uncertainty (Murray *et al.*, 2020), are likely drivers of high distress and poorer wellbeing amongst this age group. Importantly, the transition to young adulthood at age 18 generally coincides with finishing school and entering the unknown world of work, further study, or unemployment; leaving home and/or becoming financially self-sufficient; and having a strong focus on establishing intimate relationships. These stressors then gradually diminish for those who successfully transition into productive and healthy adults.

### Limitations & future directions

Extending from our study there is a need to replicate these results, both in Australia and internationally, and investigate further the risk and protective factors that may be associated with these trends, and the associations between the CMH states and other indicators of harm (e.g., self-harm, substance use, suicidality, health-risk behaviours) and productivity (including educational attainment, civic engagement). However, the current findings highlight the importance of interventions to promote wellbeing in adolescence and young adulthood in addition to services addressing mental ill-health.

The present study provides the first national community report of young people’s CMH in Australia and provides a unique profile of 12–25-year-olds captured in 2018, 2020 and 2022. However, several limitations must be acknowledged. First, while the K10 (Kessler *et al.*, 2002) is a robust and widely used indicator of psychological distress it does not provide a specific diagnosis of mental illness and as such is an indicator of mental health risk. Different outcomes may be obtained using clinical diagnostic measures or interviews which provide a more accurate categorisation of mental illness.

Second, while a quota sampling strategy was used to obtain a representative sample based on age group, gender, and state/territory distribution, this would not provide a representative sample on other criteria. Specifically, the headspace National Youth Mental Health survey reports 12–17% of young people identifying as non-heterosexual which is higher than the 6.1% reported by other available population estimates, and only 8–10% as born overseas in contrast to the 25% reported by government population estimates (Australian Institute of Health & Welfare, 2021). These differences may in part explain the higher than expected reports of psychological distress in our sample; however, further investigation of the differences by sociodemographic groups is needed. The samples also may not generalise to all young Australians as they reflect the characteristics of people who agree to join research panels or agree to participate in research when contacted by phone. The sample is then also likely to miss more vulnerable young people, including those with insecure accommodation and income. Despite large samples overall, the resultant sample size within some of the classification groups, specifically Languishing, Struggling, Floundering and Stumbling, is too small to be able to draw strong conclusions regarding age and gender differences and these trends should be considered with caution.

Third, the study is limited by the cross-sectional nature of the repeated measures which permit examination of population-level trends but which preclude an examination of within-person changes over time. Therefore, it would be of interest for a longitudinal design to discriminate more closely age/developmental from cohort differences. Finally, a potential limitation is acknowledged in relation to the CATI methodology utilised for data collection and the potential for social desirability in responses. Research comparing differences in participant responses across questionnaire delivery modes (e.g., Hoebel *et al.*, 2014) has found CATI techniques to be associated with higher rates of people indicating ‘never’ on subjective indicators of poor mental health and lower reports of poor wellbeing. This has the potential to inflate the proportion of individuals identified as flourishing. Future research should examine differences in the proportions of young people reported within each of the CMH states by mode of data collection to assess the accuracy of findings.

Given the potential complexity of individual states within each CMH category, further research also should consider the specific aspects of subjective, psychological and social wellbeing, and psychological distress that drive these classifications. Is it the experience (for Flourishing) or lack of experience (for Languishing) of the same or different wellbeing characteristics that drive these classifications? For example, is the experience of environmental mastery almost every day (as a component of eudemonic wellbeing; Ryff *et al.*, 2021) a common indicator reported by Flourishers and, conversely, the lack of experience of environmental mastery a common indicator reported by Languishers? Or is the experience of mastery a common indicator reported by Flourishers, but lack of autonomy a particular characteristic of Languishers? Determining which wellbeing characteristics reflect flourishing and languishing status is an important question to elucidate given the broad range of wellbeing indicators proposed. Equally, not all indicators will be valued as important for all respondents. Some individuals will prioritise some indicators over others. By further examining item level differences in responses, we may gain a more nuanced understanding of the elements of mental health and wellbeing that may be declining with age and gain a greater understanding of those individuals who are, for example, Struggling (reporting high wellbeing alongside mental illness).

## Conclusions

The present study found a large proportion young Australians are reportedly Flourishing, that is, experiencing high wellbeing and a lack of mental illness. While declines in Flourishing are observed as adolescents transcend into early adulthood, it remains the largest wellbeing group. Utilising indicators of positive mental wellbeing provides an important contribution to understanding the functioning of today’s youth; however, further work is required to enhance our knowledge of what it means at an individual level to be Flourishing, Middling, Languishing, Struggling, Stumbling and Floundering.

## Data Availability

The data that support the findings of this study are available from the National Youth Mental Health Foundation or via the authors, upon reasonable request.
